# Safety and Efficacy of Adding a Single Low Dose of Primaquine to the Treatment of Adult Patients With *Plasmodium falciparum* Malaria in Senegal, to Reduce Gametocyte Carriage: A Randomized Controlled Trial

**DOI:** 10.1093/cid/cix355

**Published:** 2017-06-12

**Authors:** Roger C. Tine, Khadime Sylla, Babacar T. Faye, Eugenie Poirot, Fatou B. Fall, Doudou Sow, Duolao Wang, Magatte Ndiaye, Jean Louis Ndiaye, Babacar Faye, Brian Greenwood, Oumar Gaye, Paul Milligan

**Affiliations:** 1 Department of Medical Parasitology, Faculty of Medicine, University Cheikh Anta Diop, Dakar, Senegal;; 2 Malaria Elimination Initiative, Global Health Group, University of California, San Francisco;; 3 National Malaria Control Programme, Ministère de la Santé et de l’Action sociale, Dakar, Senegal;; 4 Liverpool School of Tropical Medicine,; 5 Faculty of Infectious and Tropical Diseases, and; 6 Faculty of Epidemiology and Public Health, London School of Hygiene and Tropical Medicine, London, United Kingdom.

**Keywords:** primaquine, plasmodium, hemoglobin, safety, Senegal

## Abstract

**Introduction:**

More information is needed about the safety of low-dose primaquine in populations where G6PD deficiency is common.

**Methods:**

Adults with *Plasmodium falciparum* malaria were randomized to receive 1 of 3 artemisinin combination therapies (ACTs) with or without primaquine (0.25 mg/kg). Glucose-6-phosphate dehydrogenase (G6PD) status was determined using a rapid test. Patients were followed for 28 days to record hemoglobin concentration, adverse events, and gametocyte carriage. The primary end point was the change in Hb at day 7.

**Results:**

In sum, 274 patients were randomized, 139 received an ACT alone, and 135 received an ACT + primaquine. The mean reduction in Hb at day 7 was similar in each group, a difference in the ACT + PQ versus the ACT alone group of −0.04 g/dL (95% confidence interval [CI] −0.23, 0.31), but the effect of primaquine differed according to G6PD status. In G6PD-deficient patients the drop in Hb was 0.63 g/dL (95% CI 0.03, 1.24) greater in those who received primaquine than in those who received an ACT alone. In G6PD-normal patients, the reduction in Hb was 0.22 g/dL (95% CI −0.08, 0.52) less in those who received primaquine (interaction *P* = .01). One G6PD normal patient who received primaquine developed moderately severe anaemia (Hb < 8 g/dL). Dark urine was more frequent in patients who received primaquine. Primaquine was associated with a 73% (95% CI 24–90) reduction in gametocyte carriage (*P* = .013).

**Conclusion:**

Primaquine substantially reduced gametocyte carriage. However, the fall in Hb concentration at day 7 was greater in G6PD-deficient patients who received primaquine than in those who did not and one patient who received primaquine developed moderately severe anemia.

**Clinical Trial registration:**

PACTR201411000937373 (www.pactr.org)

## Summary

Adding low-dose primaquine to malaria treatment reduced gametocyte carriage by 73%. Patients who received primaquine had more frequent hemoglobinuria and there was a greater reduction in haemoglobin concentration in G6PD-deficient patients. One patient who received primaquine developed moderately severe anemia.

Progress in the fight against malaria [1] has led many malaria-endemic countries to outline a vision for malaria elimination [2, 3]. The World Health Organization (WHO) recommends the addition of a single low-dose of primaquine (0.25 mg/kg) to artemisinin combination therapy (ACT) for treatment of uncomplicated *Plasmodium falciparum* malaria as a component of pre-elimination or elimination programs [4]. Primaquine has been used for over 60 years in treating *Plasmodium vivax* and as a *P. falciparum* gametocytocide [5]. Primaquine rapidly kills mature gametocytes and could contribute to the reduction of malaria transmission [6]. However, countries in sub-Saharan Africa have been reluctant to use primaquine due to a lack of evidence about safety of the low-dose regimen in individuals with glucose-6-phosphate dehydrogenase (G6PD) deficiency [6]. Primaquine at higher doses causes acute haemolytic anaemia in G6PD-deficient patients [7], but there is limited information about the safety of the lower dose in populations where G6PD deficiency is common. In sub-Saharan Africa, G6PD deficiency is usually due to the G6PD (A-) allele [8]. The A- G6PD variant is associated with about 12% of normal enzymatic activity. Estimates of the frequency of this mutation in sub-Saharan Africa range from 5% to 25% [9, 10] but its frequency has been underestimated [11]. The G6PD A- phenotype is caused by the mutation A376G in the presence of 1 of 4 other mutations: G202A, G680T, T968C, or 542T, but some studies considered only the 376G/202A mutation. The 202 mutation is relatively uncommon in Senegal. In Niakhar, 12% of boys were G6PD deficient, and the 376G/968C genotype was the most prevalent [12]. In neighbouring Gambia, G6PD A- is also most commonly associated with the 968C mutation [13]. Although most individuals with the G6PD A- polymorphic variant are asymptomatic, acute hemolytic anemia can occur in heterozygous females, as well as in homozygous females and hemizygous males, under conditions of oxidative stress on red blood cells [10, 14]. This can be induced by infections and by drugs including the antimalarials primaquine and dapsone [15]. Hemolysis caused by primaquine is dose-related, and the risk is lower in patients given a single dose [16, 17] than in those given multiple doses over several days to eliminate *P.vivax* hypnozoites [18].

In studies in Mali and Burkina Faso, administration of a single dose of primaquine (0.25 mg/kg or 0.4 mg/kg) reduced gametocyte carriage and infectiousness without clinically significant haemolysis, but these studies did not include G6PD-deficient participants [19, 20]. In Tanzania, Mwaiswelo et al assessed the effect of low-dose primaquine in G6PD-deficient and normal malaria patients [21]. Three patients in each arm of the trial developed severe anemia, but none had a fall in hemoglobin concentration of more than 25%. We are not aware of any other studies in Africa.

In Senegal, elimination of malaria is planned in the central and northern parts of the country where incidence is low [22]. Introducing primaquine in these areas may help to reduce transmission, but evidence of safety is required before its use can be recommended. This study assessed the safety of adding a single fixed low-dose of primaquine (one tablet of 15 mg, corresponding to 0.25 mg/kg for a person weighing 60 kg) to the ACT regimens artemether-lumefantrine (AL), artesunate-amodiaquine (ASAQ), and dihydroartemisinine- piperaquine (DHA-PQ), when used to treat adult patients with *P. falciparum* malaria.

## METHODS

### Study Design and Population

An open-label randomized trial was undertaken in adult patients presenting with malaria at Deggo health post, Pikine, Dakar, Senegal. Patients >18 years with *P. falciparum* malaria were enrolled if they had monospecific infection with parasite density 1000–100000 trophozoites/μL and gave signed consent, and were randomized individually to receive AL, DHA-PQ, or ASAQ either alone or with primaquine. Patients were excluded if they were pregnant (confirmed by urine testing), breastfeeding, had a history of hypersensitivity to any of the study drugs, had severe malaria, moderately severe anemia (hemoglobin <8 g/dL), or had a chronic illness.

### Randomization, Treatment Allocation, and Follow-Up

Treatment assignments, prepared in permuted blocks of 18 using Stata 12 (Statacorp, College Station, Texas), were placed in numbered, sealed, opaque envelopes, which were opened in sequence by the study pharmacist at the time of treatment. The pharmacist was not involved in patient screening or assessment of outcomes. The study was not blinded, but laboratory technicians responsible for hemoglobin measurement and microscopy were unaware of treatment allocations. Fixed ACT combinations were used. For AL, tablets containing 20 mg artemether and 120 mg lumefantrine (Novartis) were given twice daily at the clinic; for DHA-PQ, patients received 3 tablets (of 40 mg dihydroartemisinin plus 320 mg piperaquine, Holley-Cotec Pharmaceuticals Co., Ltd) per day for 3 days, whereas for ASAQ, 2 tablets of 100 mg artesunate plus 270 mg amodiaquine (Sanofi-Aventis) were given per day for 3 days. One tablet of primaquine (15 mg, Sanofi-Aventis) was given to patients who were randomized to the ACT + primaquine groups. Primaquine was given at day 0 in addition to the first dose of ACT under the direct observation of the research team. After each treatment dose, participants were observed for 30 minutes and treatment readministered if the patient vomited. Patients who vomited a second time were withdrawn from the study and treated with quinine. Primaquine and AL were given with biscuits (to provide fat as recommended by the manufacturer). Clinical assessment was done on days 0, 1, and 2; subsequent visits were scheduled on days 3, 7, 14, 21, and 28. At each of these visits, hemoglobin concentration (Hb) was measured and adverse events were recorded. Adverse events, graded using a severity scale adapted from the WHO toxicity grading scale for determining the severity of adverse events, were classified as minor, moderate, or severe [23]. Urine samples were collected into clean containers at each visit and urine colour assessed by the study physician using Hillman’s chart. Grades 3–6 define an abnormally dark urine color (grade 3 and 4, moderate; grade 5 and 6, very dark urine) [24].

Hb was measured using a HemoCue machine (HemoCue® Hb 301, Angelholm, Sweden). A qualitative test of G6PD activity (CareStartTM G6PD; Access Bio. Inc., New Jersey, USA; Lot GP15J02) was done on day 0 using a finger-prick blood sample. The *CareStart* G6PD deficiency Rapid Diagnostic Test (RDT) is a qualitative enzyme chromatographic test based on the reduction of colorless nitro blue tetrazolium dye to dark-colored formazan. The sensitivity and specificity for this test range from 90% to 98% and from 87% to 96%, respectively [25, 26]. Thick and thin blood films were taken at enrollment (day 0) and on days 1, 2, 3, 7, 14, 21, and 28. Further details of laboratory methods are given in the Supplement.

### Statistical Methods

The primary endpoint was the change in Hb from day 0 to day 7. A reduction in Hb of 0.3 g/dL or less due to primaquine was considered to be small enough to be of no clinical concern. An interim analysis conducted to assess safety showed that the standard deviation of the change in Hb was 2.54 g/dL and on this basis a total sample size of 350 patients would be needed to have a 95% confidence interval that excluded a difference of 0.3 g/dL or more between groups. Secondary endpoints included change in Hb by day 3, day 14, day 21, and day 28; anemia (Hb < 11 g/dL) at any time up to day 28; clinical adverse events up to day 28, and the prevalence and density of gametocyte carriage during follow-up. The effect of primaquine on Hb was estimated using a mixed model, with time, treatment group, interaction between time and treatment, ACT regimen and gender as fixed effects, baseline Hb as a covariate, and patient as a random effect. The treatment difference at each time point, with a 95% confidence interval, was obtained from this model. A planned subgroup analysis estimated the effect of primaquine in G6PD normal and G6PD deficient patients, by fitting a model that included G6PD status, the interaction between time and G6PD status, and the interaction between treatment group and G6PD status at each time point, as fixed effects, baseline Hb as a covariate, and patient as a random effect. Wald *P*-values were obtained for testing interaction between G6PD status and treatment group at each time point, and the effect of treatment in G6PD normal and deficient patients, with a 95% confidence interval, at each time point was obtained from the model. Logistic regression was used to compare the proportion of G6PD normal and deficient patients who had a drop in Hb of 2 g/dL or more by day 7. Data were double-entered into an Access database. Analyses were conducted using Stata (StataCorp, Texas).

### Ethics

The protocol was approved by the Conseil National de Recherche en Santé in Senegal.

## RESULTS

**Figure 1.**
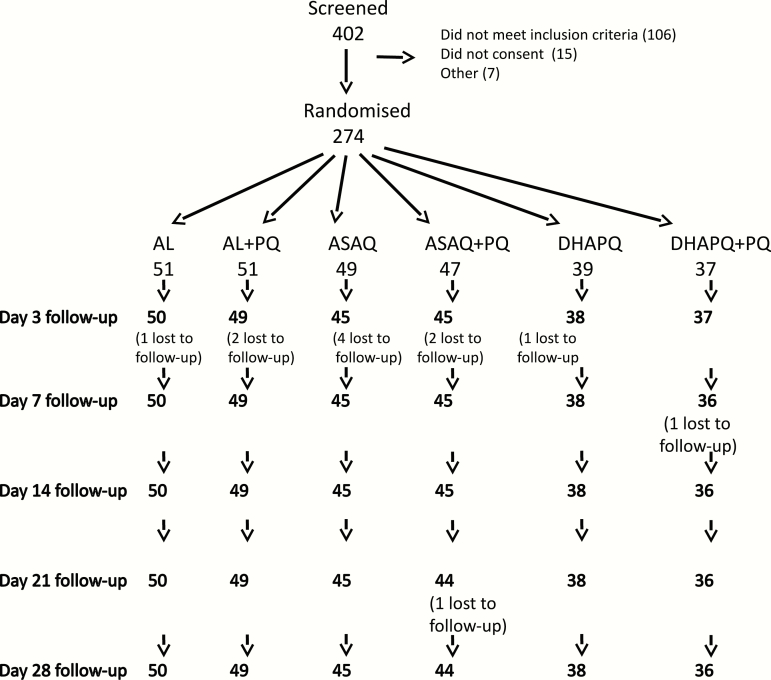
Trial profile. In sum, 128 patients were excluded. Reasons for noninclusion: 72: hemoglobin <8 g/dl; 7: intended to leave the study area; 15: did not confirm enrollment; 11: positive pregnancy test; 23: hyperparasitemia. Abbreviations: AL, artemether-lumefantrine; ASAQ, amodiaquine-artesunate; DHAPQ, dihydroartemisinin-piperaquine; PQ, primaquine.

**Figure 2.**
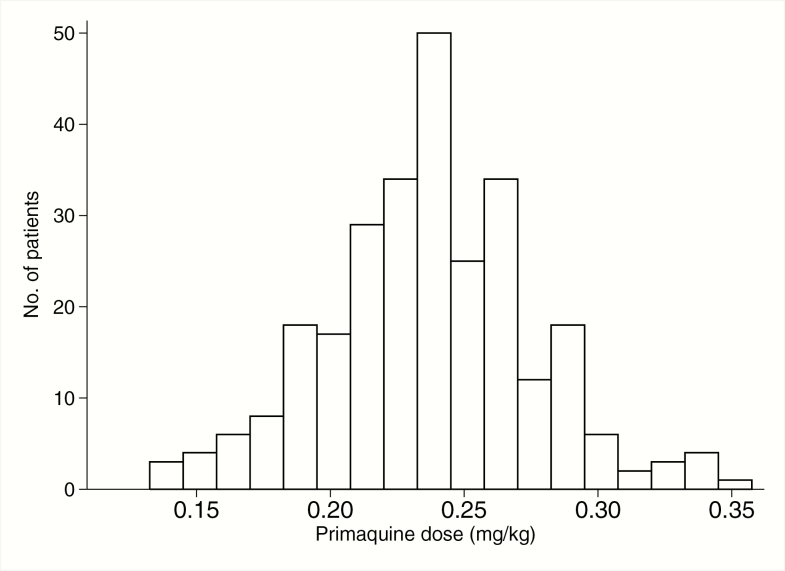
Distribution of the primaquine dose administered to study participants (in mg/kg).

During 2 malaria transmission seasons, 402 individuals with a history of fever and a positive malaria RDT were screened. The most frequent reason for exclusion was Hb < 8 g/dL at the time of screening (72 patients). In sum, 274 patients were randomized; 262 (96%) completed 28 days of follow-up (Figure 1). Recruitment was stopped at this point as the end of the transmission season had been reached, and the confidence interval on the difference in Hb by day 7 was within the prespecified range. Baseline characteristics were similar in all treatment groups (Table 1 and Table S1). The mean dose of primaquine received by patients was 0.24 mg/kg, range 0.15–0.35 (Figure 2).

**Table 1.** Baseline Characteristics of the Six Study Groups of Patients

	AL	AL + PQ	ASAQ	ASAQ + PQ	DHAPQ	DHAPQ + PQ
No. Enrolled	51	51	49	47	39	37
Male:Female	37:14	38:13	36:11	36:11	31:8	26:11
Age in years (range)	28.7 (18–74)	32.6 (18–68)	29.0 (18–74)	30.0 (18– 63)	27.1 (18– 58)	29.9 (18– 57)
Weight in kg (range)	63.7 (45–86)	67.0 (45–97)	66.7 (46–111)	65.1 (50–108)	64.9 (43–90)	63.9 (44–102)
Height in cm (range)	176.1 (166–188)	174.7 (160–195)	175.1 (156–190)	173.0 (156–188)	174.6 (154–189)	177.4 (161–188)
BMI	20.6 (12.7–27.7)	22.0 (16.2–33.6)	21.9 (14.5–44.4)	21.8 (16.4–34.1)	21.0 (8.7–31.9)	20.3 (13.7–29.2)
BMI < 18	24%	7.8%	18%	17%	21%	24%
G6PD deficient	16%	20%	20%	30%	15%	16%
Geometric mean parasite density/μL (range)	11 805 (1240–63 590)	12 347 (1000–77 612)	13 933 (1003–91 421)	12 088 (1029–61 520)	13 848 (1163–78 130)	14 938 (1200–65 718)
Hb concentration g/dL	13.1 (8.9–18.4)	13.4 (8.9–18.0)	13.9 (9.6–17.7)	13.4 (8.7–18.2)	13.4 (9.2–18.2)	13.2 (8.8–16.7)
Anemia (Hb < 11 g/dL)	12%	10%	6.1%	13%	18%	11%

Abbreviations: AL, artemether-lumefantrine; ASAQ, amodiaquine-artesunate; BMI, body mass index; DHAPQ, dihydroartemisinin-piperaquine; PQ, primaquine.

### Impact of Primaquine Treatment on Hb

The mean Hb on day 7 was similar in patients who received ACT alone and those who received ACT + primaquine. The difference (ACT + PQ−ACT alone) adjusted for baseline was −0.23 g/dL (95% CI −0.68, 0.21) for AL, −0.02 (−0.49, 0.45) for ASAQ and +0.54 (0.03, 1.06) for DHA-PQ. As the *P*-value for the interaction of ACT type with primaquine was not significant (0.0728, Table 2), for subsequent analyses the estimates of the effect of primaquine assumed that the effect was constant across ACT types. The pooled estimate of the difference in Hb on day 7 (ACT + PQ−ACT alone) was +0.06 g/dL (95% CI −0.22, 0.33; *P* = .681). Similar results were obtained at other time points. The estimates of differences at each time point obtained from the mixed model, adjusted for effects of ACT type, sex, and baseline Hb, are shown in Table 3.

**Table 2.** Mean Change in Hemoglobin Concentration From Day 0 to day 7

	ACT	ACT + PQ	Mean Difference*(95% CI)	*P*-value
AL				
Day 0	13.1	13.4	…	…
Day 7	11.9	11.9	−0.23 (−0.68, 0.21)	.306
ASAQ				
Day 0	13.9	13.4	…	…
Day 7	12.1	11.8	−0.02 (−0.49, 0.45)	.931
DHAP				
Day 0	13.4	13.2	…	…
Day 7	11.7	12.2	0.54 (0.03, 1.06)	.040
Total				
Day 0	13.5	13.4	…	…
Day 7	11.9	11.9	0.06 (−0.22, 0.33)	.681

Abbreviations: ACT, artemisinin combination therapy; AL, artemether-lumefantrine; ASAQ, amodiaquine-artesunate; CI, confidence interval; DHAP, dihydroartemisinin; PQ, primaquine.

*Estimated from linear regression comparing day 7 concentration in each group, adjusted for Hemoglobin on day 0 as a covariate. *P*-values not adjusted for multiplicity of comparisons. A test of the interaction between ACT type and PQ gave *P* = .0728. A more detailed version of this table is provided in Table S2, Supplement.

**Table 3.** Mean Hemoglobin Concentration at Each Time Point in Each Treatment Group, the Mean Change From Baseline, and the Difference Between Groups in the Change From Baseline Estimated From the Mixed Model

Day	Mean Hemoglobin Concentration (SD)		Change From Baseline, Mean (SD)		Adjusted Difference between Groups in Change From Baseline (95% CI)	
	ACT	ACT + PQ	ACT	ACT + PQ	(ACT + PQ) − (ACT)	*P*-Value
0	13.5 (1.82)	13.4 (1.94)	…	…	…	…
3	12.0 (1.65)	12.0 (1.76)	−1.49 (1.35)	−1.33 (1.32)	0.10 (−0.17, 0.37)	.474
7	11.9 (1.43)	11.9 (1.65)	−1.54 (1.35)	−1.46 (1.47)	0.04 (−0.23, 0.31)	.764
14	12.3 (1.49)	12.3 (1.31)	−1.12 (1.66)	−1.06 (1.60)	0.00 (−0.27, 0.27)	.996
21	12.8 (1.35)	12.7 (1.24)	−0.70 (1.62)	−0.61 (1.50)	0.03 (−0.24, 0.30)	.809
28	13.0 (1.28)	13.1 (1.19)	−0.49 (1.60)	−0.30 (1.77)	0.14 (−0.13, 0.41)	.314

Abbreviations: ACT, artemisinin combination therapy; CI, confidence interval; PQ, primaquine; SD, standard deviation.

### Effect of Primaquine Treatment in G6PD-Deficient and Normal Participants

**Figure 3.**
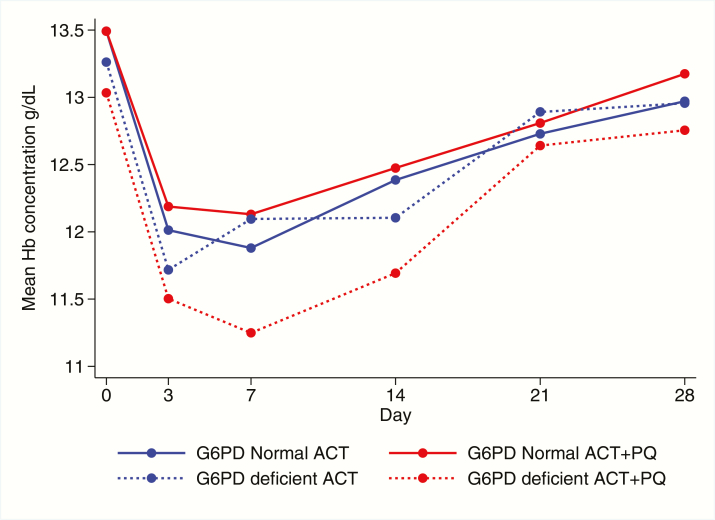
Mean hemoglobin concentration in G6PD deficient and normal patients who received primaquine or ACT alone. Abbreviations: ACT, artemisinin combination therapy; PQ, primaquine.

**Figure 4.**
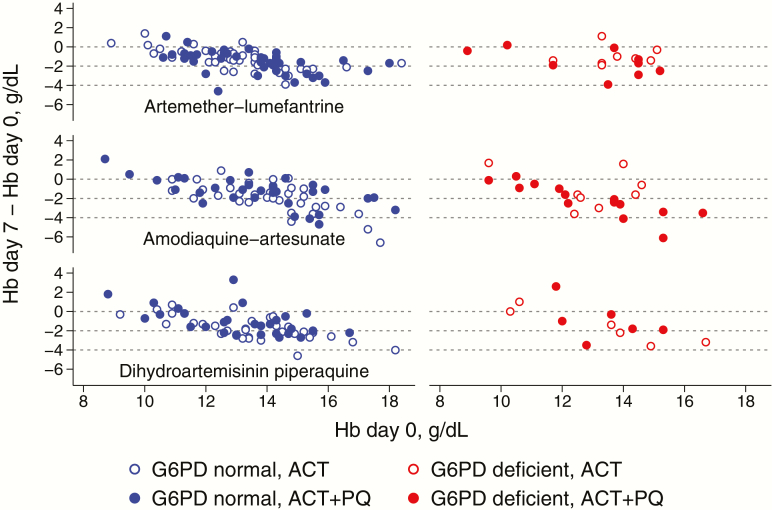
Change in hemoglobin concentration by day 7 (Hb day 7-day 0) plotted against the concentration at baseline, for each group. Abbreviations: ACT, artemisinin combination therapy; PQ, primaquine.

Fifty-four patients (20%) were G6PD-deficient; in this subgroup, the mean Hb on day 7 was 0.63 g/dL (95% CI 0.03, 1.24) lower in those who received primaquine than in those who received ACT alone (Table 4). In G6PD-normal patients, there was a slight increase in Hb on day 7 of 0.22 g/dL (95% CI −0.08, 0.52) in those who received primaquine compared with those who did not. A test of interaction between primaquine and G6PD status gave a *P*-value of .01. There was no evidence of interaction on other days (Table 4). The mean Hb in G6PD-deficient and normal patients is shown for each time point in Figure 3. Figure 4 shows the change in Hb by day 7 plotted for individual patients against their baseline value.

**Table 4.** The Mean Hemoglobin Concentration in G6PD Deficient and Normal Patients at Each Time Point, the Mean and Range of the Change From Baseline, and the Adjusted Effect of Primaquine on the Change From Baseline Estimated From the Mixed Effects Model

Day	G6PD	Mean Hb Concentration g/dL (SD)		Change From Baseline Mean (range)		Adjusted Difference in Change From Baseline (95% CI)	
	Status	ACT	ACT + PQ	ACT	ACT + PQ	(ACT + PQ) − (ACT)	*P*-Value^a^
0	Normal	13.5 (1.86)	13.5 (1.95)	…	…	…	…
	Deficient	13.3 (1.66)	13.0 (1.91)				
3	Normal	12.0 (1.64)	12.2 (1.76)	−1.47 (−6.4, +4.0)	−1.31 (−4.0, +2.1)	0.15 (−0.15, 0.45)	.544
	Deficient	11.7 (1.71)	11.5 (1.70)	−1.62 (−4.8, +0.6)	−1.50 (−4.5, +1.3)	0.00 (−0.61, 0.61)	
7	Normal	11.9 (1.37)	12.1 (1.66)	−1.60 (−6.6, +1.4)	−1.36 (−4.7, +3.3)	0.22 (−0.08, 0.52)	.010
	Deficient	12.1 (1.68)	11.2 (1.59)	−1.24 (−3.6, +1.7)	−1.75 (−6.1, +2.6)	−0.63 (−1.24, −0.03)	
14	Normal	12.4 (1.52)	12.5 (1.29)	−1.09 (−5.9, +6.0)	−1.00 (−4.8, +4.0)	0.07 (−0.23, 0.38)	.344
	Deficient	12.1 (1.35)	11.7 (1.21)	−1.23 (−3.6, +2.7)	−1.31 (−4.2, +2.1)	−0.20 (−0.81, 0.41)	
21	Normal	12.7 (1.36)	12.8 (1.31)	−0.75 (−5.6, +3.6)	−0.70 (−4.2, +3.8)	0.04 (−0.26, 0.34)	.694
	Deficient	12.9 (1.35)	12.6 (1.03)	−0.45 (−3.3, +4.7)	−0.36 (−2.9, +2.4)	−0.04 (−0.65, 0.57)	
28	Normal	13.0 (1.33)	13.2 (1.26)	−0.51 (−5.0, +4.6)	−0.32 (−3.7, +4.8)	0.18 (−0.13, 0.48)	.625
	Deficient	13.0 (1.05)	12.8 (0.87)	−0.38 (−2.8, +3.9)	−0.24 (−3.3, +3.0)	0.01 (−0.60, 0.62)	

Abbreviations: ACT, artemisinin combination therapy; CI, confidence interval; PQ, primaquine; SD, standard deviation.

^a^ The *P*-value is from a test of interaction between G6PD status and treatment.

Among G6PD-normal patients, the proportion with a drop in Hb of 2 g/dL or more at day 7 was 29% in the ACT group versus 24% in the ACT plus primaquine group, a risk ratio (RR) of 0.85 (95% CI 0.69, 1.04) (Table S3). Among G6PD-deficient patients, 26% had a drop in Hb of 2 g/dL or more after treatment with ACT by day 7 compared with 31% after treatment with ACT plus primaquine (RR = 1.19, 95% CI 0.80, 1.76).

### Anemia After Treatment With Primaquine

The proportion of patients with moderate anaemia (Hb < 11 g/dL) during the 28 day follow-up period was similar in those who received primaquine and those who received ACT alone, and there was no evidence of an interaction with G6PD status (Table S4). One patient presented with moderately severe anemia (Hb < 8 g/dL), a man in the AL + primaquine group, whose baseline Hb was 12 g/dL and who was G6PD-normal. He received AL and one tablet of primaquine (0.22 mg/kg). His Hb was 8.4 g/dL on day 3 and 7.3 g/dL on day 7 (a 39% reduction from baseline) but with no clinical features of anemia. After supplementation with iron and folate, his Hb recovered to 11.2 g/dL by day 28.

### Other Adverse Events

The incidence of adverse events was similar in all treatment groups (Table S5) except for the occurrence of dark-coloured urine, which was more common in patients who received primaquine and occurred during the first 3 days after the start of treatment; 79/135 (59%) of patients who received primaquine had dark urine (grade 3 or above) on day 1 after treatment versus 46/139 (33%) in those who received ACT alone (RR = 1.8, 95% CI 1.3, 2.3; *P* < .001). The estimates on day 2 were 25% and 12% (RR = 2.1, 95% CI 1.2–3.6; *P* = .007), and 4.5% and 1.5% (RR = 3.0, 95% CI 0.62–15; *P* = .148) on day 3. Those who had dark urine (grade 3 or above) on day 3 had a lower Hb on day 3 than those who did not (1.42 g/dL, 95% CI 1.3, 1.6; *P* = .0393).

### Parasitological Findings

**Figure 5.**
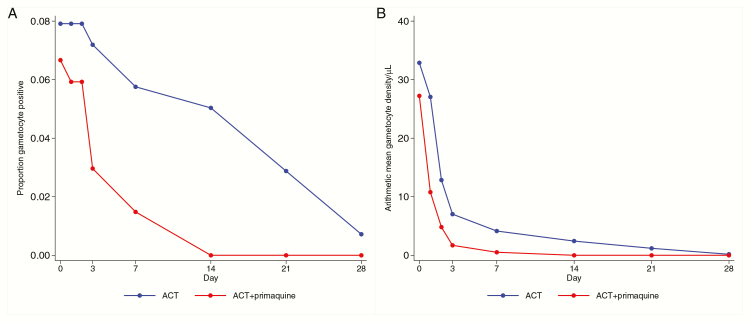
Gametocyte carriage over 28 days after treatment. *A*, Prevalence of gametocyte carriage. *B*, Arithmetic mean gametocyte density. Abbreviation: ACT, artemisinin combination therapy.

The prevalence and density of gametocyte carriage were substantially lower in patients who received primaquine than in those who did not (Figure 5). The average duration of gametocyte carriage was 1.08 days in those who received ACT alone compared to 0.29 days in the ACT + primaquine group, a reduction of 73% (95% CI 24%, 90%; *P* = .013). The area-under-the-curve of gametocyte density over time was 106.7 gametocyte-days in the ACT group and 29.5 gametocyte-days in the ACT + primaquine group, a reduction of 72% (95% CI 16%, 91%; *P* = .024, Table 5).

**Table 5.** Gametocyte Carriage at Baseline and After Treatment^a^

Day	ACT (N = 139)				ACT + PQ (N = 135)			
	No. Positive	Prevalence	Gametocyte Density		No. Positive	Prevalence	Gametocyte Density	
			Arithmetic Mean	Geometric Mean			Arithmetic Mean	Geometric Mean
0	11	7.9%	32.9	347	9	6.7%	27.2	357
1	11	7.9%	27.1	260	8	5.9%	10.8	160
2	11	7.9%	12.9	107	8	5.9%	4.8	64
3	10	7.2%	7.0	59	4	3.0%	1.7	46
7	8	5.8%	4.1	51	2	1.5%	0.5	32
14	7	5.0%	2.4	39	0	0.0%	0.0	…
21	4	2.9%	1.2	18	0	0.0%	0.0	…
28	1	0.7%	0.2	24	0	0.0%	0.0	…
Area under the curve		1.08	106.7			0.29	29.5	

Abbreviations: ACT, artemisinin combination therapy; PQ, primaquine.

^a^The mean duration of gametocyte carriage was estimated as the area under the curve of gametocyte prevalence over time, i.e., ∑d_ij_/n_i_ where n_i_ is the number of patients in group i and d_ij_ = ∑w_t_x_tij_, where x_tij_ is the gametocyte status (0 or 1) at time t in patient j in group i, and the weightings w_t_ are 0.25, 1, 1, 2.5, 5.5, 7, 10.5, and 7 for follow-up time points at 0, 1, 2, 3, 7, 14, 21, and 28 days. This is equivalent to plotting for each patient the gametocyte status (0 or 1) against time, connecting the points with straight lines, calculating the area under the resulting line, and then taking the mean area in each treatment group. The percentage efficacy against gametocyte carriage was calculated as 100×(1-R) where R is the ratio of the mean duration in the two treatment groups estimated using Poisson regression of d_ij_ on treatment group, using a robust standard error to calculate confidence intervals and *P*-values. A similar analysis was done, replacing the value of x_tij_ with the gametocyte density in patient j in group i at time t, in order to estimate the effect of treatment on gametocyte density during the 28-day follow-up.

## DISCUSSION

In 2012, the WHO revised the previously recommended primaquine dose of 0.75 mg/kg to one of 0.25 mg/kg when used together with an ACT as a pre-elimination strategy [27]. However, primaquine is not often used in sub-Saharan African countries due to safety concerns, particularly in people with G6PD deficiency [18]. In our study, one patient, who received primaquine, developed moderately severe anemia (39% reduction from baseline). His hemoglobin recovered by day 28 without any other adverse clinical findings. Overall, mean Hb on day 7 after treatment was similar in primaquine and ACT alone groups, but there was an interaction with G6PD status. In sum, 20% of patients were G6PD deficient and in this group, mean Hb on day 7 was lower if they received primaquine by 0.63 g/dL, compared to G6PD deficient patients who received ACT alone. There was no evidence of a difference on day 3, or on and after day 14. Patients who received primaquine were more likely to have dark urine on days 1 to 3 after treatment, associated with a greater drop in Hb by day 3. This resolved by day 7. None of the patients with dark urine developed severe anemia or other clinically important symptoms. Primaquine is known to cause dark urine after multiple doses at higher doses [28], but we have shown that this symptom can also occur with the lower dose of 0.25 mg/kg.

We are aware of only one other trial has investigated safety of low-dose primaquine in G6PD deficient patients in Africa [21]. In that study, in Tanzania, children and adults were enrolled, and, as in our study, patients whose baseline Hb was less than 8 g/dL were excluded; 15% of patients were phenotypically G6PD deficient. A slightly greater fall in Hb by day 7 was reported in patients who received primaquine and were G6PD deficient, but no test of interaction was performed. Analyses were also performed based on G6PD genotype, but typing was limited to the 376/202 mutation. No patients had clinically important reductions in Hb concentration. Hemoglobinuria was more frequent among G6PD-deficient patients treated with primaquine [21]. Repeated doses of 0.25 mg/kg primaquine in addition to DHA-PQ during mass drug administration campaigns in healthy adults in Thailand did not induced clinically significant hemolysis in G6PD normal or deficient individuals [29].

In our study, addition of low-dose primaquine to ACT treatment in adult patients resulted in a substantial reduction in gametocyte carriage. Reduced gametocyte carriage was also observed in studies in G6PD-normal patients in Mali and Burkina Faso [19, 20]. Thus, low-dose primaquine can substantially reduce infectiousness of malaria patients by killing *P. falciparum* gametocytes with a high degree of efficacy, and it can do this in a single dose [19, 20, 30, 31].

In our trial, patients with Hb < 8 g/dL were not enrolled. Under routine conditions, and especially during mass drug treatment studies, it may not be feasible to assess Hb prior to treatment for all individuals, and it would be difficult to predict how safe the drug would be in patients with lower Hb. In this trial, direct measurement of hemolysis was not possible. Measuring bilirubin, haptoglobin, methemoglobin, and reticulocyte count would be useful to determine whether the hemoglobin reduction is due to hemolysis or other factors.

Primaquine has become a key tool for malaria elimination [32]. This study provides additional evidence that the currently recommended single dose of primaquine (0.25 mg/kg) is well tolerated. However, the way in which primaquine should be delivered in elimination settings is still under debate. Strategies under consideration for primaquine use include adding primaquine to the treatment of symptomatic cases; identifying and treating all malaria cases in mass screen and treat (MSaT) programs; or using primaquine as a component of mass drug administration (MDA) programs [33]. Treating only symptomatic cases seen at health facilities may not have a substantial effect on transmission as these cases may represent only a small fraction of the infectious reservoir [34, 35]. MSaT and MDA have the potential substantially to reduce malaria transmission [36]. However, these strategies require drugs to be given to people who are not apparently ill and may not have malaria infection [37]. Further studies are therefore needed in order to determine the tolerability and acceptability of low-dose primaquine in MDA programs, and in patients with Hb lower than 8 g/dL. High coverage and adherence, essential for MDA programs to be effective, rely on good acceptability and tolerability of the drugs used [37]. The safety of primaquine among vulnerable groups such as pregnant woman and young children has not been well studied, but excluding these groups would limit the effectiveness of mass drug campaigns. Additional safety data on vulnerable groups may be needed to support the use of primaquine in elimination programs in Africa.

## Supplementary Data

Supplementary materials are available at *Clinical Infectious Diseases* online. Consisting of data provided by the authors to benefit the reader, the posted materials are not copyedited and are the sole responsibility of the authors, so questions or comments should be addressed to the corresponding author.

## Supplementary Material

r_tine_supplementary_information_pm_rev

## Notes

***Authors’ contribution*.** R. C. T., P. M., B. G., E. P., and O. G. conceived and designed the study. RCT, K. S., and B. T. F. supervised the data collection. P. M., D. W., and R. C. T. analyzed the data. R. C. T. was responsible of the first draft of the manuscript. All authors read and approved the final manuscript.

***Acknowledgments*.** Authors are grateful to the population of Deggo health post for their diligent help during this study. We are grateful to the malaria and elimination initiative, global health group for the technical support provided during the conception of the study. The trial was registered on the Pan African Clinical Trial Registry (www.pactr.org) database PACTR201411000937373.

***Financial support*.** This work was supported by the Malaria Capacity Development Consortium (MCDC) which is funded by the Wellcome Trust (grant nWT084289MA) and the Bill & Melinda Gates Foundation (grant 51941).

***Potential conflicts of interest*.** The authors have no conflicts of interest concerning the work reported in this paper. All authors have submitted the ICMJE Form for Disclosure of Potential Conflicts of Interest. Conflicts that the editors consider relevant to the content of the manuscript have been disclosed.
