# Supplementary material for: Safety and Efficacy of Adding a Single Low Dose of Primaquine to the Treatment of Adult Patients With Plasmodium falciparum Malaria in Senegal, to Reduce Gametocyte Carriage: A Randomized Controlled Trial
Source: Clin Infect Dis. 2017 Jun 12;65(4):535–43. doi: 10.1093/cid/cix355 (PMC5848230; doi:10.1093/cid/cix355)
Supplement: r_tine_supplementary_information_pm_rev [file cix355_suppl_r_tine_supplementary_information_pm_rev.doc]

**Supplementary information:** Safety and efficacy of adding a single low dose of primaquine to the treatment of adult patients with *Plasmodium falciparum* malaria in Senegal, to reduce gametocyte carriage: a randomized controlled trial

Roger CK Tine, Khadime Sylla, Duolao Wang, Babacar T. Faye, Eugenie Poirot, Doudou Sow, Magatte Ndiaye, Jean L Ndiaye, Babacar Faye, Brian Greenwood, Oumar Gaye, Paul Milligan.

Laboratory methods

Figure S1: Haemoglobin concentration for each G6PD-normal and deficient patient, at each time point.

Figure S2 : Mean haemoglobin concentration in G6PD deficient and normal males and females, who received primaquine or ACT alone.

Table S1 : Baseline characteristics of trial participants who received ACT alone and those who received ACT+primaquine.

Table S2: Effects of ACT type and primaquine, on haemoglobin concentration on day 7.

Table S3: Percentage of patients with a drop of haemoglobin concentration of 2g/dL or more by day 7.

Table S4: Percentage of patients with moderate anaemia (Hb<11g/dL) at each time point:

Table S5: Reported symptoms at any time by treatment arm.

Table S5a : Reported symptoms at any time, in G6PD-deficient patients

Table S5b : Reported symptoms at any time, in G6PD-normal patients

Table S6: Comparison of reported symptoms by treatment arm over the follow-up period. The value shown are the number of patients with the symptom (%).

Table S6a: Reported symptoms by treatment arm over the follow-up period among G6PD deficient participants. The values shown are the number of patients with the symptom (%).

Table S6b: Reported symptoms by treatment arm over the follow-up period among G6PD normal participants. The values shown are the number of patients with the symptom (%).

**Laboratory methods:**

Thick smears were used to determine parasite density, thin smears to determine the parasite species. Blood smears were stained with Giemsa and read by two laboratory technicians. If there was a discrepancy, a third reader was involved and the observation from the most senior reader was considered definitive. Asexual parasite density was determined by counting the number of asexual parasites per 200 white blood cells and converting to the number of parasites/µL assuming a white cell count of 8,000 per µL. Absence of malaria parasites in 200 high power fields on the thick blood film was considered as negative.

Figure S1: Haemoglobin concentration for each G6PD-normal and deficient patient, at each time point.

Figure S2 : Mean haemoglobin concentration in G6PD deficient and normal males and females, who received primaquine or ACT alone.

Table S1 : Baseline characteristics of trial participants who received ACT alone and those who received ACT+primaquine.

| **Variable** | **ACT alone** | **ACT plus primaquine** |
| --- | --- | --- |
| Number of participants | 139 | 135 |
| Age in years, mean (SD) | 28.3 (11.1) | 30.9 (11.9) |
| Sex ratio (M:F) | 3:1 | 3:1 |
| Weight in kg, mean (SD) | 65.1 (11.3) | 65.5 (11.8) |
| Height in metres, mean (SD) | 175.3 (7.1) | 174.8 (7.3) |
| Body mass index, mean (SD) | 21.2 (4.5) | 21.5 (4.0) |
| Underweight (BMI<18.5), n (%) | 33 (23.7) | 31 (22.9) |
| G6PD deficient, n (%) | 24 (17.3) | 30 (22.2) |
| Parasite density/µL, median (IQR) | 15,766 (6760–32,061) | 15,180 (6680–31,615) |
| Haemoglobin g/dL, mean (SD) | 13.5 (1.8) | 13.4 (1.9) |
| Anaemia (Hb<11g/dl) , n (%) | 16 (11.5) | 16 (11.9) |

Table S2: Effects of ACT type and primaquine, on haemoglobin concentration on day 7. The values in the table are from regression analysis of the day 7 concentrations, with baseline Hb as a covariate, and ACT type, primaquine and their interaction as covariates in the model. (Mean Hb on day 7 was greater by 0.54 g/dL in those who received DHAPQ+PQ compared to those that received DHAP alone, etc.)

| Variable | Difference (95%CI) | P-value | Difference (95%CI) | P-value |
| --- | --- | --- | --- | --- |
| Baseline Hb g/dL | 0.57 (0.50,0.64) | <0.001 |  |  |
|  |  |  |  |  |
| AL | Reference |  |  |  |
| AL+PQ | -0.23 (-0.68,0.21) | 0.306 |  |  |
| ASAQ | Reference |  |  |  |
| ASAQ+PQ | -0.02 (-0.49,0.45) | 0.931 |  |  |
| DHAP | Reference |  |  |  |
| DHAP+PQ | 0.54 (0.03,1.06) | 0.040 |  |  |
|  |  |  |  |  |
| ACT alone: |  |  |  |  |
| AL | Reference |  |  |  |
| ASAQ | -0.21 (-0.67,0.24) | 0.358 | Reference |  |
| DHAP | -0.34 (-0.82,0.14) | 0.161 | -0.12 (0.62,0.36) | 0.612 |
|  |  |  |  |  |
| ACT+PQ: |  |  |  |  |
| AL | Reference |  |  |  |
| ASAQ | 0.00 (-0.46,0.45) | 0.989 | Reference |  |
| DHAP | 0.43 (-0.05,0.92) | 0.081 | 0.44 (-0.06,0.93) | 0.085 |

(P-values not adjusted for multiplicity). Interaction P-value 0.0728

Table S3: Percentage of patients with a drop of haemoglobin concentration of 2g/dL or more by day 7.

|  | G6PD normal | G6PD deficient |
| --- | --- | --- |
| ACT alone | 28.5% | 26.1% |
| ACT + Primaquine | 24.2% | 31.0% |
| Risk ratio (95%CI) | 0.85 (0.69,1.04) | 1.19 (0.80,1.76) |

*Interaction p-value: 0.133*

**Table S4: Percentage of patients with moderate anaemia (Hb<11g/dL) at each time point:**

|  |  |  | Number (%) of patients with Hb<11g/dL | | | | | |
| --- | --- | --- | --- | --- | --- | --- | --- | --- |
|  |  | No.  enrolled | Day 0 | Day 3 | Day 7 | Day 14 | Day 21 | Day 28 |
| G6PD normal | ACT | 115 | 13 (11%) | 27 (23%) | 35 (30%) | 19 (17%) | 12 (10%) | 9 (8%) |
|  | ACT+PQ | 105 | 10 (9.5%) | 23 (22%) | 24 (23%) | 11 (10%) | 7 (7%) | 2 (2%) |
| G6PD deficient | ACT | 24 | 3 (12.5%) | 8 (33%) | 6 (25%) | 5 (21%) | 2 (8%) | 1 (4%) |
|  | ACT+PQ | 30 | 5 (17%) | 11 (37%) | 14 (47%) | 9 (30%) | 1 (3%) | 1 (3%) |

(One patient had severe anaemia (Hb<8g/dL), a G6PD-normal patient in the ACT+primaquine group on day 7)

Table S5: Reported symptoms at any time by treatment arm.

| Sign | Total  (N=274) | ACT  (N= 139) | ACT+PQ  (N= 135) | p value |
| --- | --- | --- | --- | --- |
| Asthenia | 46 (17%) | 25 (17.8%) | 21 (16%) | 0.59 |
| Dizziness | 56 (20%) | 34 (25%) | 22 (16%) | 0.09 |
| Abdominal pain | 47 (17%) | 26 (19%) | 21 (16%) | 0.49 |
| Nausea | 29 (11%) | 11 (7.9%) | 18 (13%) | 0.14 |
| Vomiting (day1) | 7 (2.5%) | 04 (2.9%) | 3 (2.2%) | 0.73 |
| Diarrhoea | 7 (2.5%) | 04 (2.9%) | 3 (2.2%) | 0.73 |
| Pallor | 63 (23%) | 28 (20%) | 35 (26%) | 0.25 |
| Rash | 4 (1.5%) | 2 (1.4%) | 02 (1.5%) | 0.98 |
| Cyanosis | 0 (0%) | 0 (0%) | 0 (0%) | - |
| Dark urine* | 126 (46%) | 46 (33%) | 80 (59%) | <0.001 |
| Grade 3 | 98 (36%) | 38 (27%) | 60 (44%) | 0.003 |
| Grade 4 | 45 (16%) | 11 (7.9%) | 34 (25%) | <0.001 |
| Grade 5 | 4 (1.5%) | 1 (0.7%) | 3 (2.2) | 0.30 |
| Grade 6 | 15 (5.5%) | 6 (4.3%) | 9 (6.7%) | 0.39 |

**Dark urine colour was defined as grade 3,4,5 and 6 according to the Hillman grading chart.*

| Table S5a : Reported symptoms at any time, in G6PD-deficient patients | | | |
| --- | --- | --- | --- |
| Sign | Total | ACT | ACT+PQ |
|  | (N=54) | (N= 24) | (N= 30) |
| Asthenia | 7 (13%) | 5 (21%) | 2 (6.7%) |
| Dizziness | 8 (15%) | 5 (21%) | 3 (10%) |
| Abdominal pain | 9 (17%) | 4 (17%) | 5 (17%) |
| Nausea | 5 (9.3%) | 1 (4.2%) | 4 (13%) |
| Vomiting (day1) | 0 (0%) | 0 (0%) | 0 (0%) |
| Diarrhoea | 2 (3.7%) | 2 (8.3%) | 0 (0%) |
| Pallor | 13 (24%) | 3 (13%) | 10 (33%) |
| Rash | 1 (1.8%) | 0 (0%) | 1 (3.3%) |
| Cyanosis | 0 (0%) | 0 (0%) | 0 (0%) |
| Dark urine* | 25 (46%) | 8 (33%) | 17 (57%) |
| Grade 3 | 17 (32%) | 5 (21%) | 12 (40%) |
| Grade 4 | 11 (20%) | 5 (21%) | 6 (20%) |
| Grade 5 | 1 (1.8%) | 0 (0%) | 1 (3.3%) |
| Grade 6 | 5 (9.3%) | 2 (8.3%) | 3 (10%) |

| Table S5b : Reported symptoms at any time, in G6PD-normal patients | | | |
| --- | --- | --- | --- |
| Sign | Total | ACT | ACT+PQ |
|  | (N=220) | (N= 115) | (N= 105) |
| Asthenia | 34 (18%) | 20 (17%) | 19 (18%) |
| Dizziness | 48 (22%) | 29 (25%) | 19 (18%) |
| Abdominal pain | 38 (18%) | 22 (19%) | 16 (15%) |
| Nausea | 24 (11%) | 10 (8.7%) | 14 (13%) |
| Vomiting (day1) | 7 (3.2%) | 4 (3.5%) | 3 (2.9%) |
| Diarrhoea | 5 (2.3%) | 2 (1.7%) | 3 (2.9%) |
| Pallor | 50 (23%) | 25 (22%) | 25 (24%) |
| Rash | 3 (1.4%) | 2 (1.7%) | 1 (0.9%) |
| Cyanosis | 0 (0%) | 0 (0%) | 0 (0%) |
| Dark urine* | 143 (65%) | 62 (54%) | 81 (77%) |
| Grade 3 | 81 (37%) | 33 (29%) | 48 (46%) |
| Grade 4 | 34 (15%) | 6 (5.2%) | 28 (27%) |
| Grade 5 | 3 (1.4%) | 1 (0.8%) | 2 (1.9%) |
| Grade 6 | 10 (4.5%) | 4 (3.5%) | 6 (5.7%) |

Table S6: Comparison of reported symptoms by treatment arm over the follow-up period. The value shown are the number of patients with the symptom (%).

|  | **D1** | | **D2** | | **D3** | | | **D7** | | | **D14** | | **D21** | | **D28** | |
| --- | --- | --- | --- | --- | --- | --- | --- | --- | --- | --- | --- | --- | --- | --- | --- | --- |
|  | **ACT** | **ACT +PQ** | **ACT** | **ACT**  **+PQ** | **ACT** | **ACT**  **+PQ** | | **ACT** | **ACT+PQ** | | **ACT** | **ACT+PQ** | **ACT** | **ACT+PQ** | **ACT** | **ACT+PQ** |
| Asthenia | 12  (8.6) | 10  (7.4) | 7  (5.0) | 7  (5.2) | 4 (2.9) | 4  (3.0) | | 0  (0) | 2  (1.5) | | 0 (0) | 3  (2.2) | 1  (0.7) | 3  (2.2) | 2  (1.4) | 0 (0) |
| Dizziness | 12 (8.6) | 10 (7.4) | 8  (5.8) | 8  (5.9) | 11  (7.9) | 6  (4.4) | | 6  (4.3) | 3  (2.2) | | 8  (5.8) | 6  (4.4) | 3  (2.2) | 2  (1.5) | 4  (2.9) | 2  (1.5) |
| Abdominal pain | 20 (14.4) | 11 (8.2) | 6  (4.3) | 11  (8.2) | 1 (0.7) | 3  (2.2) | | 1  (0.7) | 4  (2.9) | | 3  (2.2) | 0  (0) | 0  (0) | 0  (0) | 0  (0) | 0  (0) |
| Nausea | 9  (6.5) | 16 (11.9) | 2  (1.4) | 2  (1.5) | 1 (0.7) | 0  (0) | | 0  (0) | 0  (0) | | 0  (0) | 1  (0.7) | 0  (0) | 0  (0) | 0  (0) | 0  (0) |
| Vomiting in first 24 hours | 3  (2.2) | 2 (1 .5) | 1  (0.7) | 0  (0) | 0  (0) | 1  (0.7) | | 0  (0) | 0  (0) | | 0  (0) | 0  (0) | 0  (0) | 0  (0) | 0  (0) | 0  (0) |
| Diarrhoea | 2  (1.4) | 2  (1.5) | 1  (0.7) | 2  (1.5) | 1  (0.7) | 0  (0) | | 0  (0) | 0  (0) | | 0  (0) | 0  (0) | 0  (0) | 0  (0) | 0  (0) | 0  (0) |
| Pallor | 15 (10.8) | 23 (17.0) | 25 (18.0) | 25 (18.5) | 17  (12.2) | | 27  (20.0) | 17  (12.2) | | 18  (13.3) | 13  (9.4) | 13  (9.6) | 9  (6.5) | 8  (5.9) | 9  (6.5) | 9  (6.7) |
| Rash | 1  (0.7) | 0  (0) | 2  (1.4) | 1  (0.7) | 0  (0) | 0  (0) | | 0  (0) | 0  (0) | | 0  (0) | 0  (0) | 0  (0) | 0  (0) | 0  (0) | 0  (0) |
| Cyanosis | 0  (0) | 0  (0) | 0  (0) | 0  (0) | 0  (0) | 0  (0) | | 0  (0) | 0  (0) | | 0  (0) | 0  (0) | 0  (0) | 0  (0) | 0  (0) | 0  (0) |
| Dark urine colour* | 46 (33.1) | 79 (58.5) | 16 (11.5) | 33 (24.4) | 2  (1.4) | 6  (4.4) | | 0  (0) | 1  (0.7) | | 0  (0) | 0  (0) | 0  (0) | 0  (0) | 0  (0) | 0  (0) |
| Grade 3 | 29 (20.9) | 40 (29.6) | 11 (7.9) | 22  (16.3) | 2  (1.4) | 5  (3.7) | | 0  (0) | 1  (0.7) | | 0  (0) | 0  (0) | 0  (0) | 0  (0) | 0  (0) | 0  (0) |
| Grade 4 | 10  (7.2) | 26 (19.3) | 4  (2.9) | 9  (6.7) | 0  (0)0 | 1  (0.7) | | 0  (0) | 0  (0) | | 0  (0) | 0  (0) | 0  (0) | 0  (0) | 0  (0) | 0  (0) |
| Grade 5 | 1  (0.7) | 2  (1.5) | 0  (0) | 1  (0.7) | 0  (0) | 0  (0) | | 0  (0) | 0  (0) | | 0  (0) | 0  (0) | 0  (0) | 0  (0) | 0  (0) | 0  (0) |
| Grade 6 | 6  (4.3) | 9  (6.7) | 1  (0.7) | 1  (0.7) | 0  (0) | 0  (0) | | 0  (0) | 0  (0) | | 0  (0) | 0  (0) | 0  (0) | 0  (0) | 0  (0) | 0  (0) |

*Dark urine colour was defined as grade 3,4,5 and 6 according to the Hillman grading chart*

| Table S6a: Reported symptoms by treatment arm over the follow-up period among G6PD deficient participants. The values shown are the number of patients with the symptom (%). | | | | | | | | | | | | | | |
| --- | --- | --- | --- | --- | --- | --- | --- | --- | --- | --- | --- | --- | --- | --- |
|  | **D1** | | **D2** | | **D3** | | **D7** | | **D14** | | **D21** | | **D28** | |
|  | **ACT** | **ACT +PQ** | **ACT** | **ACT+PQ** | **ACT** | **ACT +PQ** | **ACT** | **ACT+PQ** | **ACT** | **ACT+PQ** | **ACT** | **ACT+PQ** | **ACT** | **ACT+PQ** |
| Asthenia | 2 (8.3%) | 1 (3.4%) | 2 (8.7%) | 1 (3.4%) | 1 (4.3%) | 2 (6.9%) | 0 | 0 | 0 | 0 | 0 | 0 | 0 | 0 |
| Dizziness | 0 | 3 (10%) | 2 (8.7%) | 2 (6.9%) | 2 (8.7%) | 1 (3.4%) | 1(4.3%) | 1 (3.4%) | 0 | 1(3.4%) | 1(4.3%) | 0 | 0 | 0 |
| Abdominal pain | 2 (8.3%) | 3 (10%) | 1 (4.3%) | 3 (10%) | 0 | 1 (3.4%) | 0 | 2 (6.9%) | 0 | 0 | 0 | 0 | 0 | 0 |
| Nausea | 1 (4.2%) | 3 (10%) | 0 | 0 | 0 | 0 | 0 | 0 | 0 | 1(3.4%) | 0 | 0 | 0 | 0 |
| Vomiting in first 24 hours | 0 | 0 | 0 | 0 | 0 | 0 | 0 | 0 | 0 | 0 | 0 | 0 | 0 | 0 |
| Diarrhoea | 0 | 0 | 1 (4.3%) | 0 | 1 (4.3%) | 0 | 0 | 0 | 0 | 0 | 0 | 0 | 0 | 0 |
| Pallor | 1 (4.2%) | 6(21%) | 5 (22%) | 3 (10%) | 3 (13%) | 9 (31%) | 2(8.7%) | 6 (21%) | 0 | 5 (17%) | 0 | 3 (10%) | 0 | 2 (6.9%) |
| Rash | 0 | 0 | 0 | 0 | 0 | 0 | 0 | 0 | 0 | 0 | 0 | 0 | 0 | 0 |
| Cyanosis | 0 | 0 | 0 | 0 | 0 | 0 | 0 | 0 | 0 | 0 | 0 | 0 | 0 | 0 |
| Dark urine* | 8 (33%) | 17(57%) | 7 (30%) | 17 (58%) | 2(8.7%) | 2 (6.9%) | 0 | 0 | 0 | 0 | 0 | 0 | 0 | 0 |
| Grade 3 | 1 (4.2%) | 8 (27%) | 4 (17%) | 3 (10%) | 2 (8.7%) | 2(6.9%) | 0 | 0 | 0 | 0 | 0 | 0 | 0 | 0 |
| Grade 4 | 4 (17%) | 3 (10%) | 2 (8.7%) | 3 (10%) | 2 (8.7%) | 0 | 0 | 0 | 0 | 0 | 0 | 0 | 0 | 0 |
| Grade 5 | 0 | 1 (3.3%) | 0 | 0 | 0 | 0 | 0 | 0 | 0 | 0 | 0 | 0 | 0 | 0 |
| Grade 6 | 2 (8.3%) | 3 (10%) | 0 | 0 | 0 | 0 | 0 | 0 | 0 | 0 | 0 | 0 | 0 | 0 |
| *Dark urine colour was defined as a grade of 3,4,5 or 6 according to the Hillman grading chart* | | | | | | | | |  |  |  |  |  |  |

| Table S6b: Reported symptoms by treatment arm over the follow-up period among G6PD normal participants. The values shown are the number of patients with the symptom (%). | | | | | | | | | | | | | | |  |
| --- | --- | --- | --- | --- | --- | --- | --- | --- | --- | --- | --- | --- | --- | --- | --- |
|  | **D1** | | **D2** | | **D3** | | **D7** | | **D14** | | **D21** | | **D28** | |  |
|  | **ACT** | **ACT +PQ** | **ACT** | **ACT+PQ** | **ACT** | **ACT+PQ** | **ACT** | **ACT+PQ** | **ACT** | **ACT+PQ** | **ACT** | **ACT+PQ** | **ACT** | **ACT+PQ** |  |
| Asthenia | 10 (8.9%) | 9 (8.6%) | 5 (4.5%) | 6 (5.8%) | 3 (2.7%) | 2 (1.9%) | 0 | 2 (2.0%) | 0 | 3 (3.0%) | 1 (0.9%) | 3 (3.1%) | 2 (1.8%) | 0 |  |
| Dizziness | 12 (11%) | 7 (6.7%) | 6 (5.4%) | 6 (5.8%) | 9 (8.2%) | 5 (4.9%) | 5 (4.5%) | 2 (2.0%) | 8 (7.3%) | 5 (5.1%) | 2(1.8%) | 2 (2.0%) | 4 (3.7%) | 2 (2%) |  |
| Abdominal pain | 18 (16%) | 8 (7.7%) | 5 (4.5%) | 8 (7.7%) | 1 (0.9%) | 0 | 1 (0.9%) | 2 (2.0%) | 3 (2.7%) | 0 | 0 | 0 | 0 | 0 |  |
| Nausea | 8 (7.1%) | 13 (13%) | 2 (1.8%) | 2 (1.9%) | 1 (0.9%) | 0 | 0 | 0 | 0 | 0 | 0 | 0 | 0 | 0 |  |
| Vomiting in first 24hrs | 3 (2.7%) | 2 (1.9%) | 1 (0.9%) | 0 | 0 | 1 (0.9%) | 0 | 0 | 0 | 0 | 0 | 0 | 0 | 0 |  |
| Diarrhoea | 2 (1.8%) | 2 (1.9%) | 0 | 2 (1.9%) | 0 | 0 | 0 | 0 | 0 | 0 | 0 | 0 | 0 | 0 |  |
| Pallor | 14 (13%) | 17 (16%) | 8 (7.2%) | 11 (11%) | 14 (13%) | 18 (18%) | 15 (14%) | 12 (12%) | 13 (12%) | 8 (8.1%) | 9 (8.2%) | 5 (5.1%) | 9 (8.3%) | 7 (7%) |  |
| Rash | 1 (0.9%) | 0 | 2 (1.8%) | 1 (0.9%) | 0 | 0 | 0 | 0 | 0 | 0 | 0 | 0 | 0 | 0 |  |
| Cyanosis | 0 | 0 | 0 | 0 | 0 | 0 | 0 | 0 | 0 | 0 | 0 | 0 | 0 | 0 |  |
| Dark urine* | 62 (54%) | 80 (76%) | 46 (41%) | 74 (71%) | 2 (1.8%) | 4 (3.9%) | 1 (0.9%) | 5 (4.9%) | 0 | 0 | 0 | 0 | 0 | 0 |  |
| Grade 3 | 28 (24%) | 32 (31%) | 7 (6.2%) | 19 (18%) | 2 (1.8%) | 3 (2.9%) | 1 (0.9%) | 5 (4.9%) | 0 | 0 | 0 | 0 | 0 | 0 |  |
| Grade 4 | 6 (5.2%) | 23 (22%) | 2 (1.8%) | 6 (5.8%) | 0 | 1(0.9%) | 0 | 0 | 0 | 0 | 0 | 0 | 0 | 0 |  |
| Grade 5 | 1 (0.9%) | 1 (0.9%) | 0 | 1 (0.9%) | 0 | 0 | 0 | 0 | 0 | 0 | 0 | 0 | 0 | 0 |  |
| Grade 6 | 4 (3.5%) | 6 (5.7%) | 1 (0.9%) | 1 (0.9%) | 0 | 0 | 0 | 0 | 0 | 0 | 0 | 0 | 0 | 0 |  |
| *Dark urine colour was defined as a grade of 3,4,5 or 6 according to the Hillman grading chart* | | | | | | | | |  |  |  |  |  |  |  |
